# Development of a standardized intracranial vessel model from 3D time-of-flight magnetic resonance angiography data of the SHIP cohort

**DOI:** 10.1007/s00234-026-04147-w

**Published:** 2026-08-19

**Authors:** Antonia Dalmer, Stefan Siewert, Dominique Passler, Hagen Paetow, Christoph Brandt-Wunderlich, Daniel Cantré, Ebba Beller, Sönke Langner, Klaus-Peter Schmitz, Marc-André Weber, Felix Streckenbach

**Affiliations:** 1 Institute and Policlinic of Radiology, Pediatric Radiology and Neuroradiology, University Medical Center Rostock, Rostock, Germany; 2 Institute for ImplantTechnology and Biomaterials e.V., Rostock-Warnemünde, Germany

**Keywords:** TOF-MRA, Intracranial arteries, Three-dimensional vascular modeling, Computer-aided design (CAD), Population-based vascular model, Vessel segmentation, 3D printing

## Abstract

**Purpose:**

A standardized, population-based three-dimensional (3D) computer-aided design (CAD) model of the intracranial arterial system was developed from time-of-flight magnetic resonance angiography (TOF-MRA) data of the Study of Health in Pomerania (SHIP) cohort, tailored for realistic simulation and experimental neurovascular applications.

**Methods:**

An averaged intracranial TOF-MRA dataset generated from 4308 individual whole-body MRI examinations of the SHIP cohort was used as the anatomical basis. Intracranial arteries were segmented using *3D Slicer* with Frangi-based vessel enhancement, semi-automatic region-growing, and manual refinement to obtain continuous vascular masks. Centerlines were extracted with *VMTK (Vascular Modelling Toolkit)*, and vessel radii were computed via distance mapping. The resulting centerline and radius data were imported into a CAD environment (*Creo Parametric*) to reconstruct smooth vessel centerlines, generate circular cross-sections, and create a lofted three-dimensional lumen model, which was converted into a hollow geometry with a uniform wall thickness and exported as an STL file for 3D printing.

**Results:**

The proposed workflow yielded a geometrically consistent, hollow 3D model of the central intracranial arteries, representing a population-averaged arterial anatomy with smooth vessel courses, gradual diameter transitions, and a closed, continuous wall. The CAD model could be successfully manufactured as a physical 3D-printed phantom and provides a stable, reproducible test environment for digital and in vitro investigations of neurovascular interventions under standardized anatomical conditions.

**Conclusions:**

Population-based TOF-MRA data can be transformed into a technically robust and anatomically meaningful intracranial reference model suitable for CAD-based simulation and additive manufacturing. The resulting 3D CAD geometry serves as a reusable reference for comparative studies, methodological validation, early-stage device development, and training in endovascular neurosurgery, without aiming to replace patient-specific models.

## Key points

Time-of-flight magnetic resonance angiography (TOF-MRA)–based vascular modeling.

Population-averaged intracranial arterial anatomy.

Three-dimensional (3D) cerebrovascular reconstruction.

Image-based vessel segmentation and centerline extraction.

Computational modeling of cerebral blood vessels.

## Introduction

Cerebrovascular diseases such as ischemic stroke, intracranial aneurysms, arteriovenous malformations, and vasculitis are among the leading causes of morbidity and mortality worldwide. Magnetic resonance imaging (MRI) plays an essential role in the diagnosis and management of these conditions. In particular, three-dimensional (3D) time-of-flight magnetic resonance angiography (TOF-MRA) has become a widely adopted non-invasive imaging technique that allows for high-resolution visualization of intracranial vessels without the need for contrast agents. Its ability to delineate the vascular architecture in fine detail makes it especially valuable in the evaluation of stenoses, aneurysms, and vessel anomalies [1–4]. 

3D TOF-MRA data sets are also an essential part of whole-body MR examinations in large population-based cohort studies with the potential of generating standardized reference values for anatomical structures. The Study of Health in Pomerania (SHIP) is a prospective cohort whose subjects are recruited from the general population of the German State of Mecklenburg–Western Pomerania. The analysis of the participant’s individual anatomy is an essential part of the study [5–7]. 

In particular, understanding the vascular anatomy is critical for accurate diagnosis, preoperative planning, and interventional strategies. This is especially relevant in endovascular neurosurgery, where precise knowledge of the 3D geometry of cerebral arteries guides the selection of devices such as stents, flow diverters, or coils, and impacts clinical outcomes. Hence, angiographic imaging, both non-invasive and catheter-based, is indispensable not only for the initial diagnostic process but also for planning and guiding neurointerventional procedures [8–13]. 

Recent deep-learning-based approaches have demonstrated promising performance for automated segmentation of cerebral vessels in MRA, including attention-based and residual-network architectures. However, these studies primarily focus on image segmentation, whereas the present work aims to generate a standardized, CAD-compatible vascular reference model from population-based TOF-MRA data [14, 15]. 

In recent years, advances in medical image processing and computational modeling have enabled the transformation of raw imaging data into realistic and patient-specific 3D vascular models. Among the most widely used platforms in this context is *3D Slicer*, an open-source software for visualization and analysis of medical image data. *3D Slicer* supports semi-automated and automated segmentation of vascular structures, generation of vessel centerlines, and mesh processing tools that are crucial for the reconstruction of accurate 3D representations of cerebral vasculature [16, 17]. 

A particularly important step in this workflow is the extraction of vessel centerlines, which facilitates the quantification of vessel curvature, length, and diameter. Furthermore, centerlines enable the reformatting of complex 3D geometries into simplified two-dimensional (2D) planes aligned with the course of the vessel. This transformation is of great value for virtual intervention planning and simulation, allowing for more intuitive manipulation and analysis of devices in the vascular lumen [18, 19]. 

Given the variability of intracranial vessel anatomy across individuals, the development of standardized 3D-CAD (computer-aided design) models based on population-derived imaging data is a compelling goal. Such models offer the potential to simulate neurovascular procedures with high anatomical realism, evaluate device behavior under realistic constraints, and train clinicians in a risk-free environment [20, 21]. 

While patient-specific vascular models provide excellent anatomical fidelity for individual case planning, they are less suitable for standardized device evaluation and comparative experimental studies because geometric properties vary substantially between subjects. Likewise, probabilistic vascular atlases provide statistical information on vessel location and occurrence but are generally not intended for direct CAD reconstruction, additive manufacturing, or in vitro experimentation. The present work addresses this gap by translating a large-scale population-averaged TOF-MRA dataset into a manufacturable and reusable intracranial vascular reference geometry. The objective is not to replace patient-specific models, but rather to provide a standardized anatomical platform that allows reproducible testing, comparison of devices and methods, and training under controlled anatomical conditions.

So the primary aim of this work is to develop a methodology for generating standardized 3D-CAD models of intracranial vessels from population-based TOF-MR angiographic data. These models are designed for realistic simulation of neurovascular interventions, including virtual and physical stent placement, catheter navigation, and device testing. This includes segmentation of vascular structures, extraction of geometric centerlines, and the generation of 2D-projection planes to facilitate virtual stenting procedures. Ultimately, such a framework could serve as a powerful tool for medical education, device testing, and procedural planning in endovascular neurosurgery.

## Methods

### Study design and image data

All participants provided written informed consent prior to inclusion. The study was reviewed and approved by the Ethics Committee of University Medicine Greifswald and carried out in accordance with the principles of the Declaration of Helsinki. Separate ethics approval was not required for this subproject.

To reconstruct a 3D model of intracranial blood vessels, TOF-MRA data of the head was used. The study population was derived from the baseline examination (SHIP) of the population-based Study of Health in Pomerania (SHIP), which enrolled 4,308 participants aged 20–79 years from northeast Germany, including 2,192 women and 2,116 men. Whole-body magnetic resonance imaging was performed using a standardized protocol on a 1.5-T MRI scanner (Magnetom Avanto, Siemens Medical Systems, Erlangen, Germany). Quality assurance was ensured through standardized operating procedures, pilot testing before study initiation, semi-annual quality reports, and regular observer certification [6, 7, 22]. 

Ethical approval for this study was obtained within the framework of the SHIP study, which was reviewed and approved by the responsible institutional ethics committee.

The acquisition included a 3D slab over the circle of Willis with high in-plane resolution and thin slices to enable reliable visualization of the major intracranial arteries The underlying MRA data consisted of an averaged dataset generated from *n* = 4308 individual scans, both to capture interindividual variability in vascular anatomy and to improve the signal-to-noise ratio. To obtain the averaged intracranial TOF-MRA dataset, individual volumes were registered to a common anatomical reference space using an initial rigid alignment followed by nonlinear deformation. The registered datasets were then intensity-normalized and voxel-wise averaged, resulting in a population-based representation of intracranial vessel signal and anatomy.

**Fig. 1:**
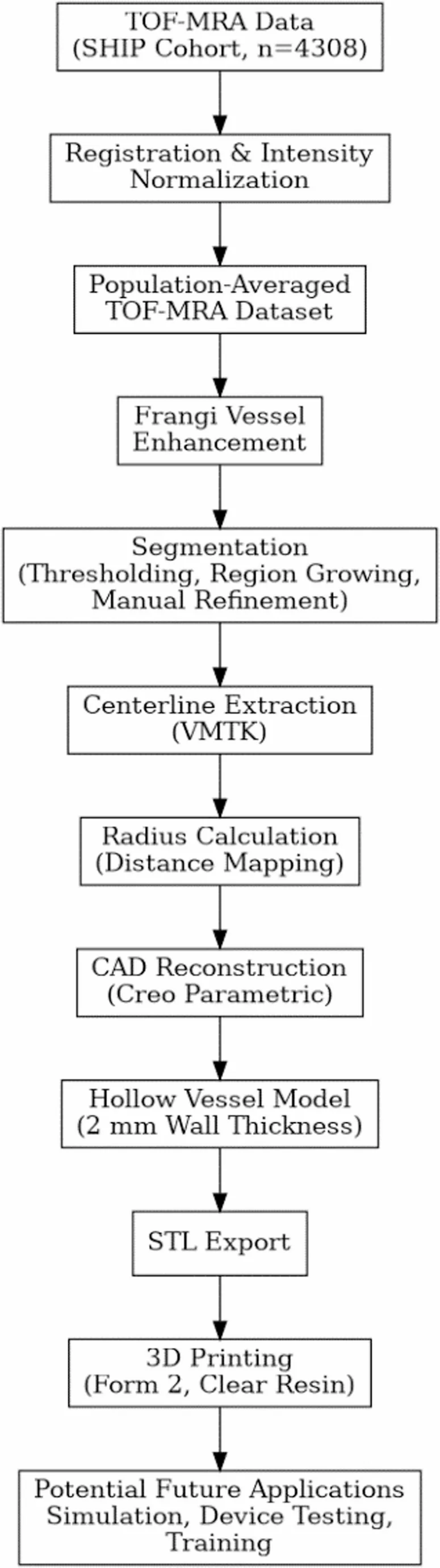
Study workflow

The study comprised four principal stages: (1) preparation and segmentation of the population-averaged TOF-MRA dataset, (2) extraction of vessel centerlines and local radii, (3) CAD-based reconstruction and generation of a hollow vessel geometry, and (4) export and additive manufacturing of the resulting model. An overview of the complete workflow, including the principal inputs, software environments, and outputs, is provided in Fig. 1.

### TOF-MRA vessel segmentation and centerline extraction

The segmentation of native TOF-MRA datasets was conducted using the open-source platform *3D Slicer* version 5.8.1 (https://www.slicer.org/). To improve the visibility and segmentation quality of the soft tissue, the Segment Editor Extra Effects was used. To ensure consistent vessel extraction across subjects, a multi-step segmentation workflow was applied.

First, each TOF-MRA dataset was visually inspected to exclude scans with severe motion artifacts or insufficient vessel-to-background contrast. Intensity normalization was performed to reduce inter-subject variability in signal levels. Subsequently, vessel enhancement was achieved using a vesselness filter based on the Frangi filter, which highlights tubular, high-intensity structures while suppressing non-vascular tissue.

**Fig. 2:**
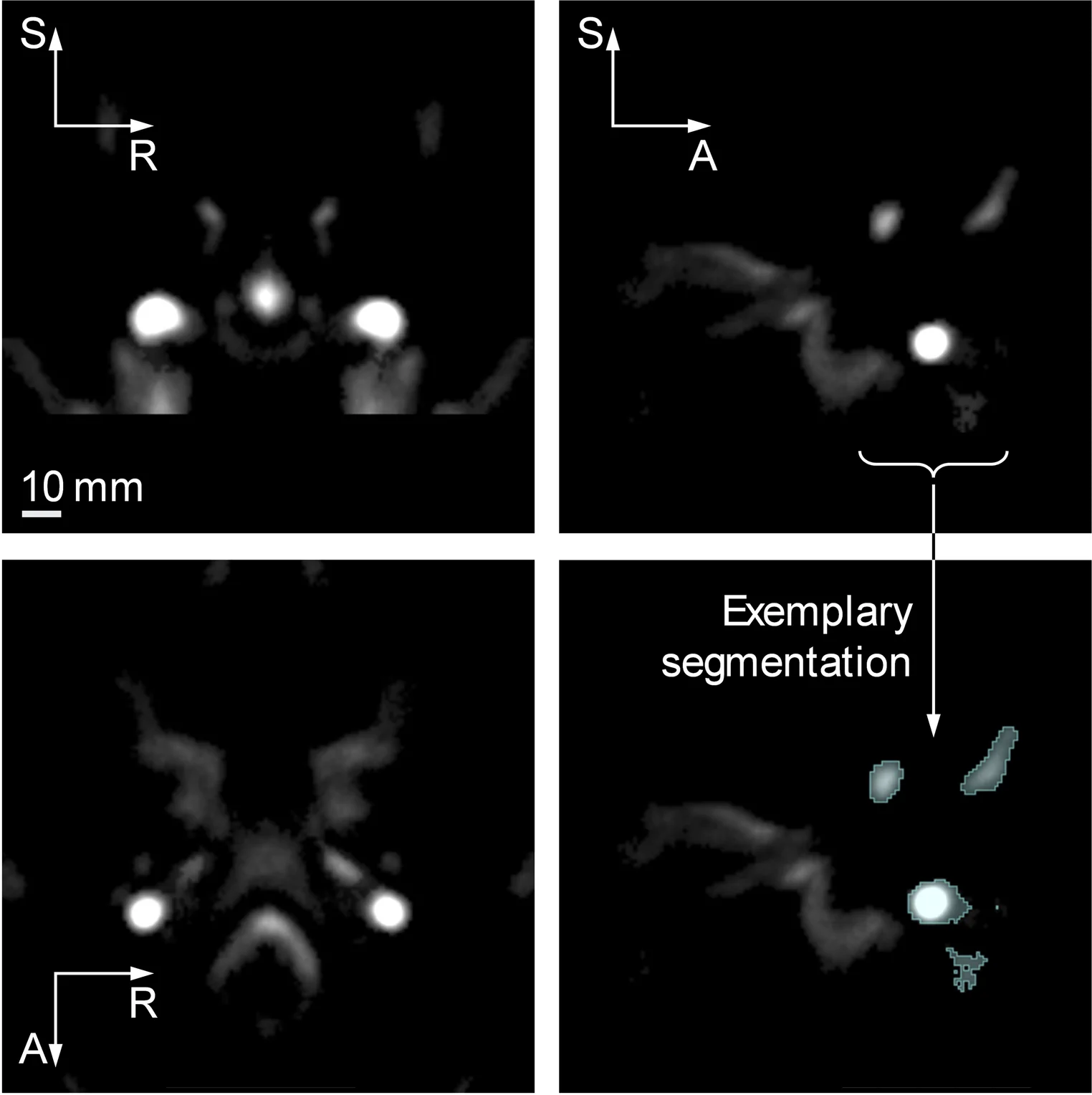
Representation of the underlying MRI data. Orthogonal sections of the TOF-MRA data set are shown in sagittal, coronal, and axial planes. Example representation of the segmented intracranial vascular structures based on the TOF-MRA data set.* S* superior, *R* right, *a* anterior

To extract the vascular structures, an island-based segmentation approach with global intensity thresholding was applied to the enhanced images. A global threshold in the normalized intensity range of 0.03 to 0.97 was empirically determined to reliably separate vascular signal from background while preserving vessel continuity. Connected vessel segments were identified and isolated, and misclassified areas or artifacts were removed manually using the Scissors tool to ensure an anatomically plausible representation of the intracranial vascular tree. An exemplary vascular anatomy is shown in Fig. 2.

The enhanced datasets were then subjected to semi-automatic segmentation, combining threshold-based region growing and morphological refinement to delineate the intracranial arterial tree. Manual correction was applied in areas where automatic methods failed to capture vessel continuity or introduced false positives, particularly near the skull base and in areas of low signal intensity.

After completion of the segmentation, the resulting vascular masks were used to generate 3D centerline models via the *VMTK (Vascular Modelling Toolkit) Centerline Extraction* module. For this, inlet and outlet points were manually defined for the respective vessel segments. This step applied a Voronoi-based thinning algorithm to compute the medial axis of the vascular structures. Centerlines were smoothed using a moderate smoothing factor and resampled at a uniform step size to balance geometric fidelity and numerical stability. At each resampled centerline point, the vessel radius was calculated as the shortest distance to the segmented vessel wall using an internal Euclidean distance transform. Each centerline represents a single vessel segment and allows for a numerical description of the vessel geometry.

The combination of vessel segmentation and centerline modeling enabled a precise geometric reconstruction of the cerebrovascular anatomy suitable for statistical analysis, atlas generation, and downstream applications such as flow simulation or device testing. To process the centerline geometries, a program developed in-house was used. This program generated points in a defined spatial grid along each centerline. Each point was assigned a local vessel radius derived from the segmented data. These resulting point and radius information served as an interface between medical image processing and CAD-based (CAD: computer-aided design) modeling.

To generate a probabilistic cerebrovascular atlas, all segmented vessel models were registered to a common anatomical reference space. Each dataset contributed to the calculation of vessel occurrence probability and local vessel diameter at the voxel level. Vessel diameters were estimated from the centerline data using distance mapping techniques. Only voxels with vessel presence in multiple subjects were included to ensure statistical robustness of the resulting diameter atlas.

The segmentation workflow was applied consistently to the averaged TOF-MRA dataset using the same processing pipeline throughout the study. Threshold values were determined empirically based on visual assessment of vessel continuity and suppression of background structures. Manual refinement was limited to removal of obvious artifacts, separation of falsely connected structures, and restoration of interrupted vessel segments where automatic segmentation was insufficient. All manual corrections were performed by a single experienced operator.

### CAD modeling and 2D projection

For simulation purposes, the 3D vessel models were further processed into CAD-compatible geometries. A 2D projection was computed by transforming the 3D vascular structures into a medically relevant planar representation. This projection step preserved critical anatomical features while enabling simplified and computationally efficient modeling for subsequent simulations.

**Fig. 3:**
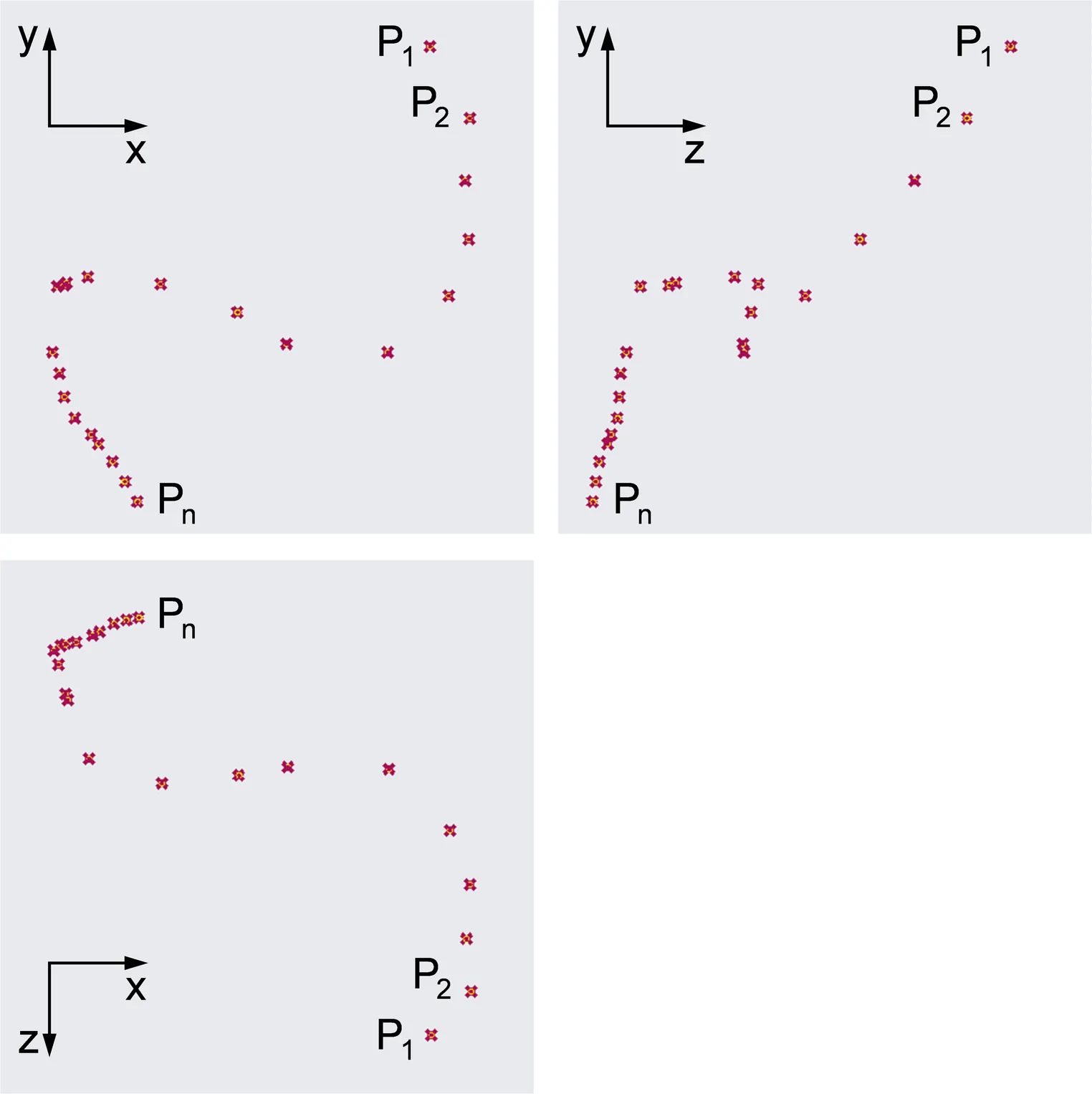
Representation of the centerline point data of an exemplary reconstructed artery imported into the CAD environment (*Creo Parametric*). Orthogonal views of the resulting point cloud are shown in three planes of the global coordinate system. The point data form the geometric basis for the subsequent reconstruction of the vessel geometry

The centerline point data generated from image processing was imported into *Creo Parametric 10* (PTC Inc., Boston, Massachusetts, USA). Separate point clouds were generated for each centerline and served as the geometric basis for the further construction of the 3D vessel models. Overall, ten point clouds were imported using the same Cartesian coordinate system. An example of an imported point cloud (P_1_, …, P_n_) is shown in Fig. 3.

**Fig. 4:**
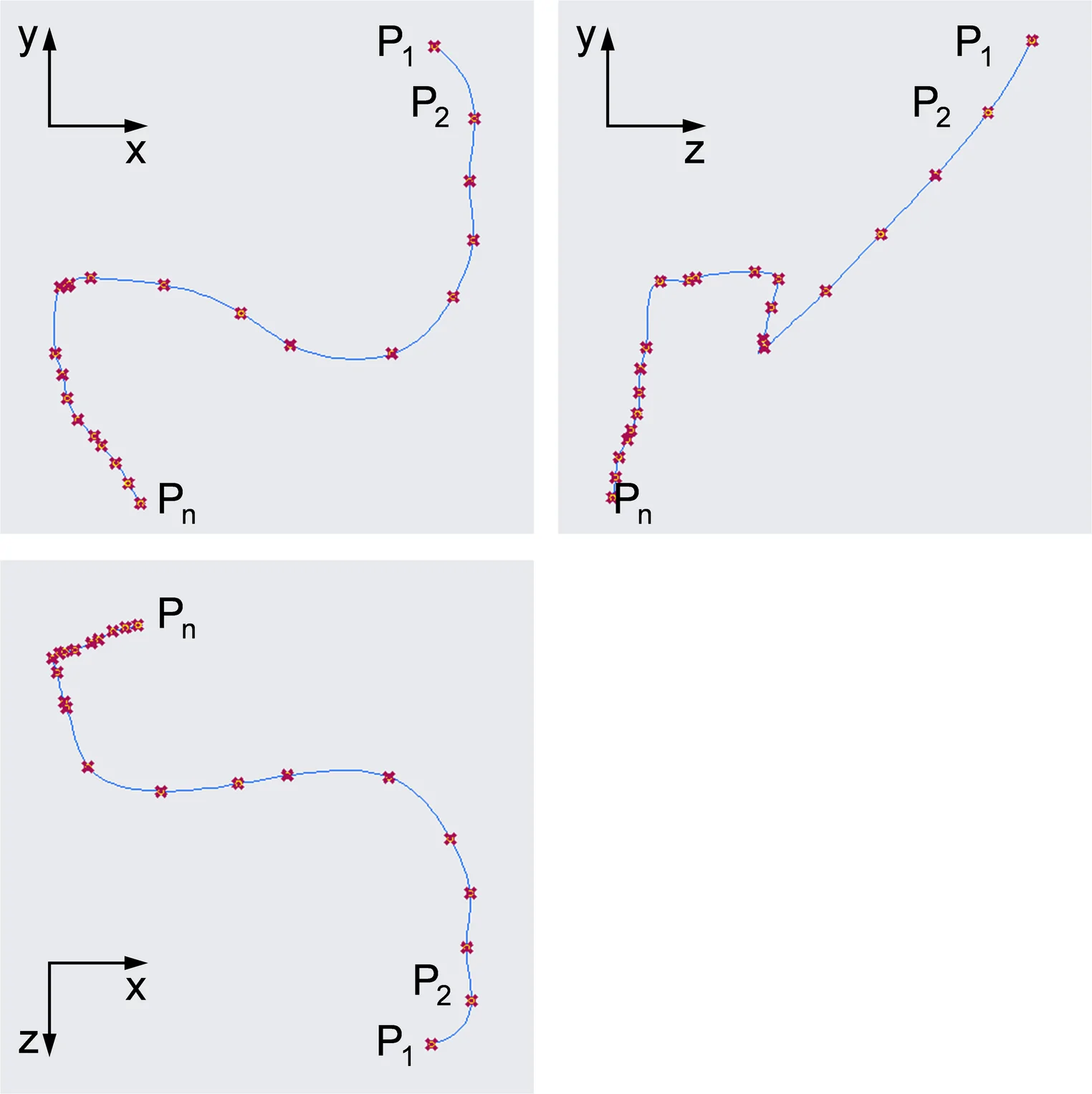
Generation of a continuous centerline from discrete point data. Orthogonal projections of an interpolating spline curve, which was adapted to the imported centerline points of an exemplary vessel segment, are shown in three planes of the global coordinate system. The spline curve represents a smooth and geometrically consistent approximation of the vessel axis

Continuous spatial curves were generated for each vessel axis using the imported point data. To do so, the discrete points were connected by an interpolating spline curve to obtain a smooth and geometrically consistent centerline (see Fig. 4).

**Fig. 5:**
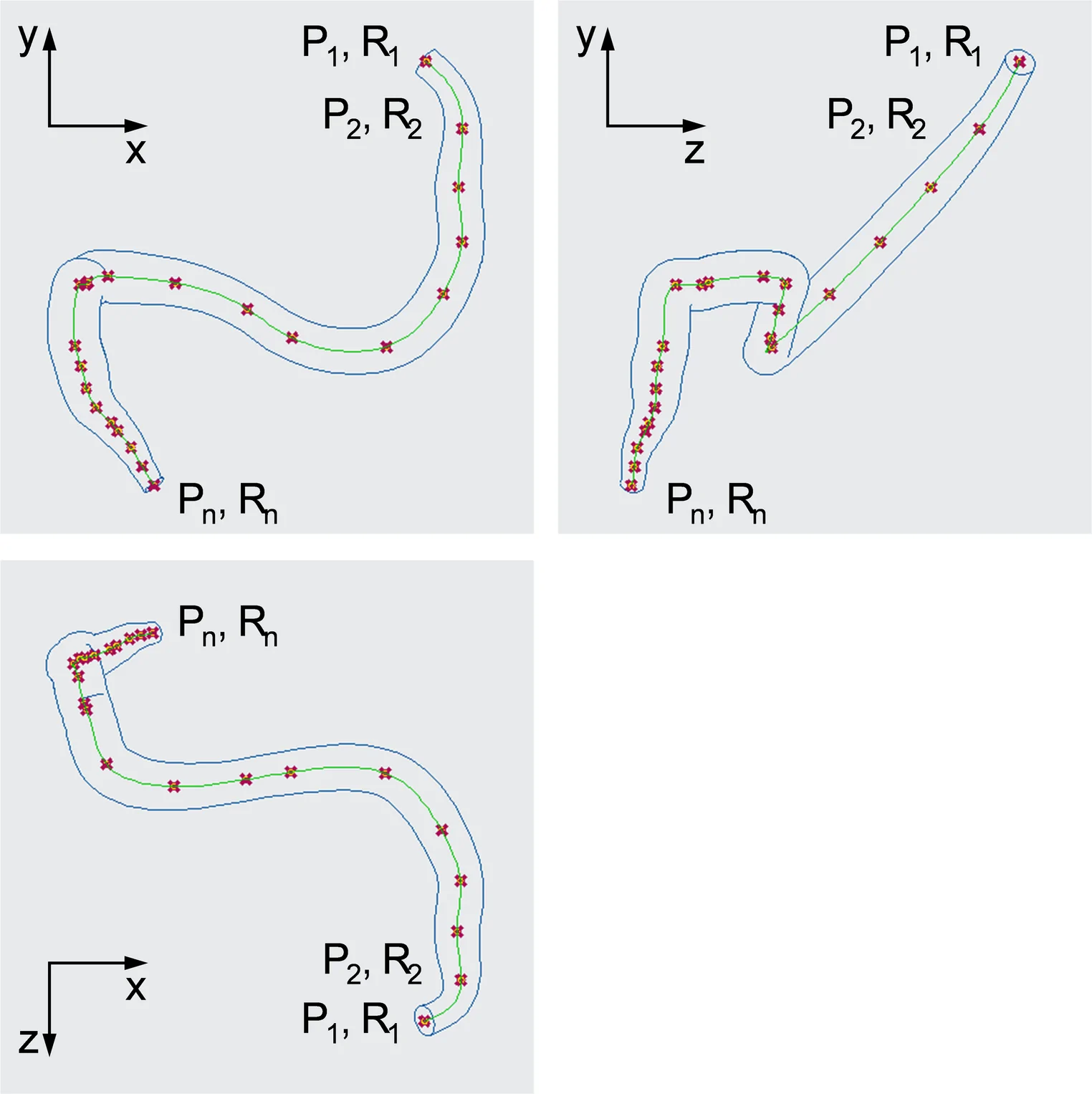
Reconstruction of the 3D vessel geometry along the centerline. Circular cross-sections were defined at the positions of the discrete points along the previously generated centerline, each aligned orthogonally to the local tangent of the centerline and with a radius corresponding to the associated vessel radius. The complete 3D vessel geometry (lumen) was then generated by a loft operation connecting the sequential cross sections along the centerline. An example of a reconstructed vessel segment is shown in three planes of the global coordinate system

Along the centerline, circular cross-sections were defined at the positions of the discrete points (P_1_, …, P_n_), each of which was perpendicular to the local tangent of the centerline. The radius (R_1_, …, R_n_) of each cross-section corresponded to the respective centerline point’s vessel radius. The complete 3D vessel geometry was generated by a loft operation that connected the sequential circular cross-sections along the centerline (see Fig. 5).

**Fig. 6:**
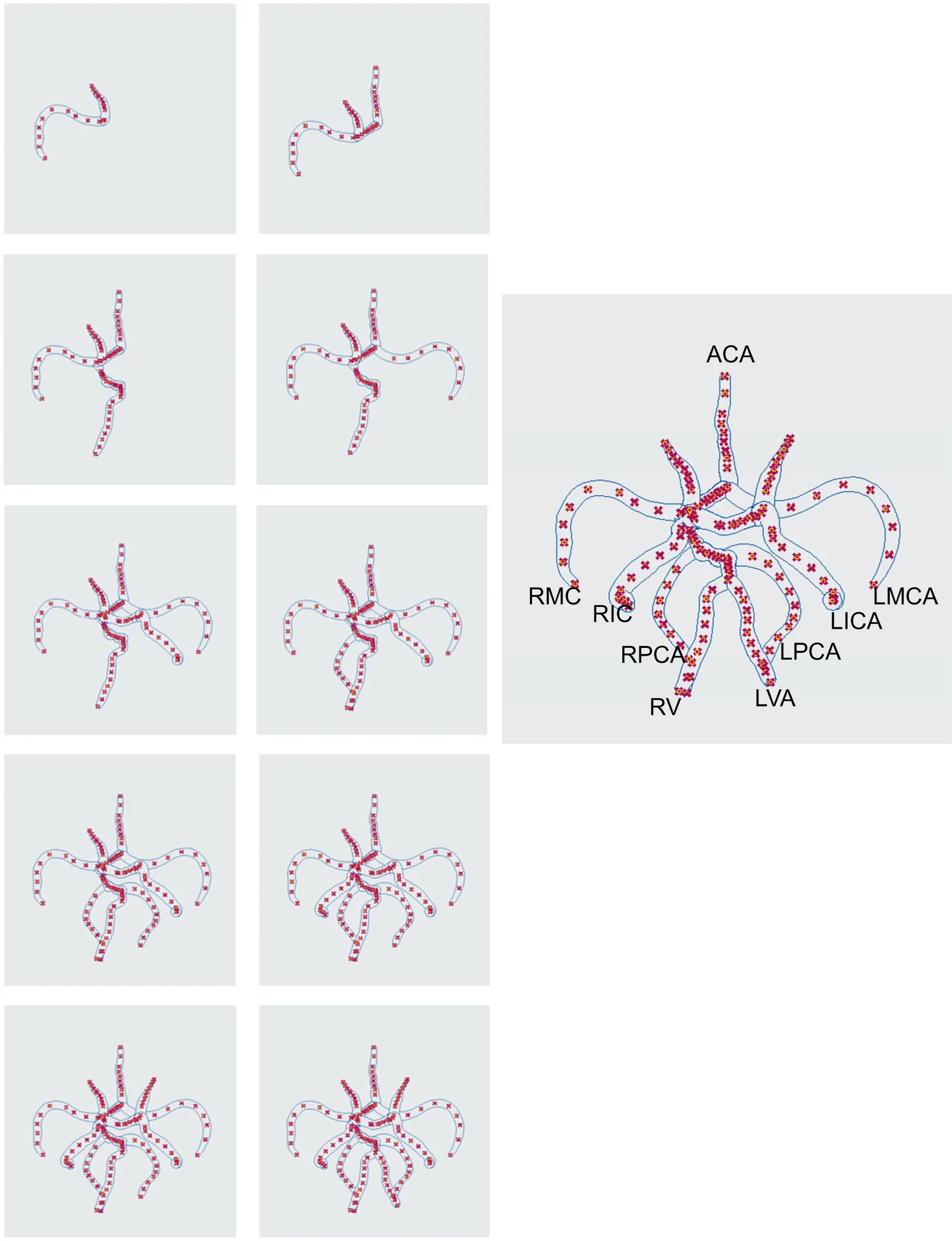
Step-by-step construction of the 3D vessel model by successively adding individual vessel segments. The partial images show successive stages of reconstruction, beginning with a single reconstructed artery and ending with the complete 3D geometry of the intracranial vessel model. Each reconstruction stage adds another vessel segment to the existing model and illustrates the modular structure of the overall geometry. *RICA* Right Internal Carotid Artery, *LICA* Left Internal Carotid Artery, *RVA* Right Vertebral Artery, *LVA* Left Vertebral Artery, *ACA* Anterior Cerebral Artery, *RPCA* Right Posterior Cerebral Artery, *LPCA* Left Posterior Cerebral Artery, *RMCA* Right Middle Cerebral Artery, *LMCA* Left Middle Cerebral Artery

Each vessel segment was modeled separately and sequentially added to the overall model (Fig. 6). The resulting volume bodies were visually and geometrically verified to avoid self-intersections, unrealistic diameter changes, or discontinuities.

For further modeling and application, only one side of the intracranial vascular system was deliberately considered. This simplification served to reduce geometric complexity and improve the clarity and manageability of the model for experimental and potential simulation-based investigations. The selected vessel side showed a fully reconstructable and geometrically consistent representation of the relevant vessel segments, while the contralateral side was not considered further due to incomplete or qualitatively limited reconstruction.

The reconstructed CAD model initially represented only the vessel lumen. To create a physically realizable model for additive manufacturing, the lumen was converted into a hollow vessel model with a defined wall thickness of 2 mm. This wall thickness was chosen as a compromise between geometric fidelity, mechanical stability, and robustness during 3D printing and handling. A radial offset operation was applied to generate an outer vessel surface with a constant wall thickness along the entire vessel path, while the original lumen remained as the inner cavity. The final closed hollow vessel model was exported in STL (stereolithography) format and 3D-printed using a Form 2 printer and Clear Resin (Formlabs Inc., Somerville, Massachusetts, USA) [23, 24]. 

## Results

**Fig. 7:**
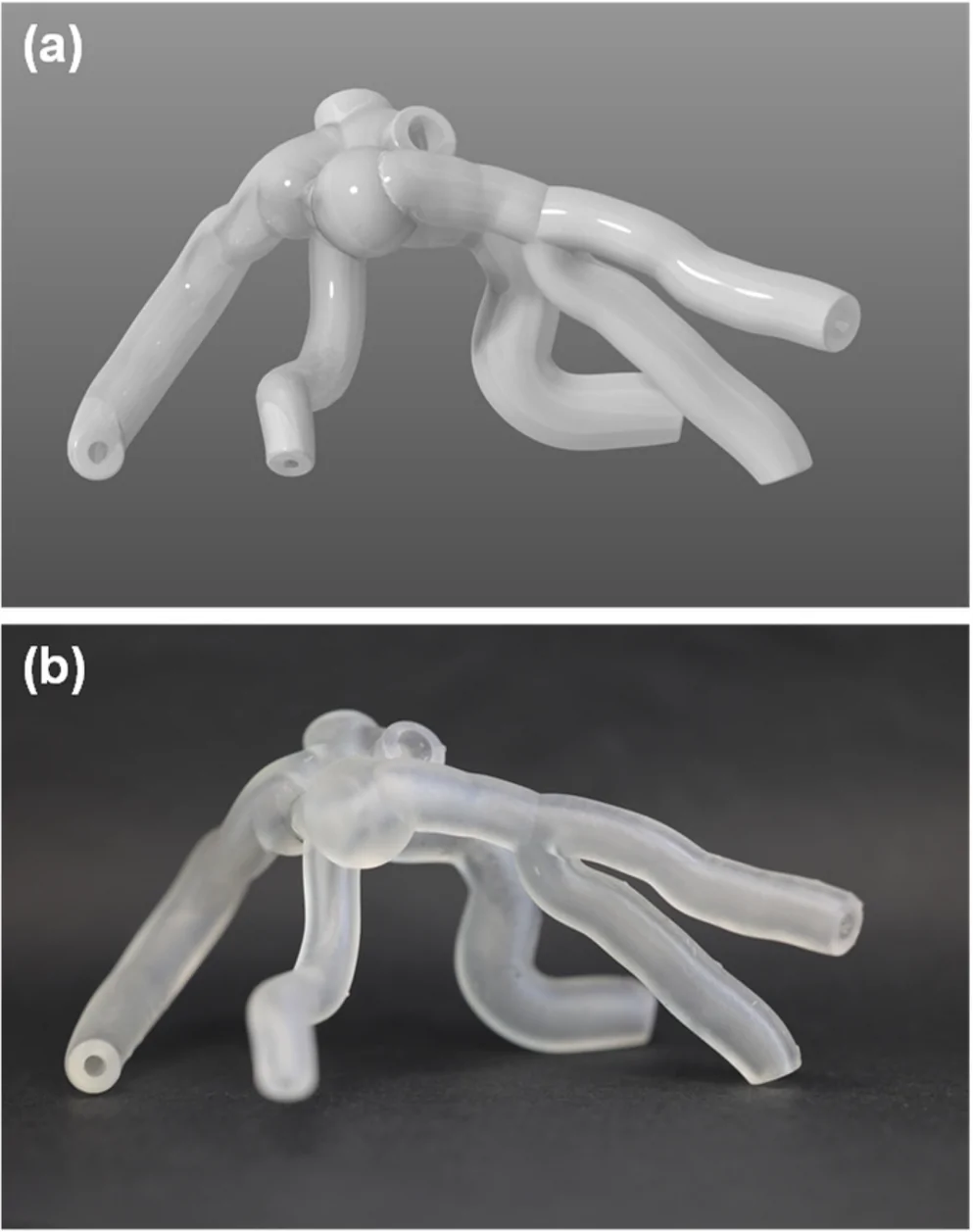
Three-dimensional hollow vessel model of the intracranial arteries: (**a**) virtual CAD model and (**b**) physical 3D printed model

Based on the described workflow, a 3D, hollow vessel model of the intracranial arteries could be successfully reconstructed (see Fig. 7). The resulting model realistically depicts the anatomical geometry of the central intracranial vessel sections and has a continuous, closed vessel wall with a defined wall thickness.

Quantitative geometric characteristics of all reconstructed vessel segments are summarized in Table 1. For each vessel segment, the number of reconstructed control points together with minimum, maximum, and mean vessel radius as well as curvature and torsion values were calculated directly from the extracted centerlines. These parameters provide an objective description of the resulting reference geometry and serve as a basis for future comparative investigations.

**Table 1:** Geometric characteristics of the reconstructed vessel segments

vessel	control point no. []	curve length [mm]	radius [mm]			curvature [1/mm]		curvature radius [mm]		torsion [1/mm]	
			min	max	mean	mean	max	mean	max	mean	max
ACA	33	35,55	2,98	4,66	3,48	0,36	99,31	2,79	0,01	0,03	1,12
LMCA	79	82,32	2,32	5,29	3,99	0,27	18,92	3,66	0,05	0,02	0,71
LICA	52	55,97	4,57	6,01	5,22	0,27	29,44	3,65	0,03	0,02	0,42
LPCA	52	54,04	2,52	4,96	3,23	0,34	4,05	2,93	0,25	0,02	0,12
LVA	20	23,18	1,10	6,01	3,52	0,35	13,13	2,84	0,08	0,03	0,64
RVA	45	46,38	2,93	4,60	4,12	0,25	3,64	4,03	0,28	0,01	0,07
RPCA	56	59,57	3,51	6,06	5,18	0,27	11,26	3,76	0,09	0,02	0,17
RICA	51	53,82	1,97	3,79	2,82	0,35	14,54	2,88	0,07	0,02	0,53
RMCA	65	65,94	2,80	4,79	4,02	0,27	2,85	3,71	0,35	0,02	0,08

The reconstructed model includes the major proximal segments of the intracranial arterial tree, encompassing the intracranial internal carotid artery, the proximal middle cerebral artery (M1 and initial M2 segments), the anterior cerebral artery (A1 and A2 segments), as well as the basilar artery and selected proximal posterior circulation branches. The vessel courses exhibit smooth curvature transitions without self-intersections or abrupt diameter changes, as verified by visual and geometric inspection of the CAD geometry. The total length of the reconstructed vessel centerlines and the total lumen volume can be quantified directly from the CAD model and provide a basis for further standardized geometric analyses. The reconstructed centerline network enables direct extraction of quantitative geometric descriptors. Table 1 summarizes the principal characteristics of each reconstructed vessel segment, including the number of control points, vessel radius, curvature, and torsion. Together, these parameters objectively describe the resulting standardized vascular geometry and demonstrate the geometric consistency of the reconstruction.

The presented results show that a geometrically consistent, hollow vessel model can be generated from clinical TOF-MRA data, which can be used digitally and physically. The resulting vessel model provides a geometrically consistent test environment suitable for in vitro investigations of neurovascular interventions.

In addition, the reconstructed vessel model does not represent an individual patient anatomy but rather reflects an averaged anatomical configuration derived from a large population-based cohort. By using an aggregated TOF-MRA dataset composed of 4308 individual scans, interindividual anatomical variability was reduced, allowing for the extraction of stable and reproducible vascular trajectories. The resulting geometry therefore represents typical morphological characteristics of intracranial arteries without emphasizing extreme anatomical variants or subject-specific features.

The geometry derived from the aggregated centerline and diameter information exhibits smooth, continuous vessel courses with moderate curvature and gradual diameter transitions along the vessel axes. Local irregularities commonly observed in individual segmentations were largely mitigated through population-based averaging. As a result, the reconstructed vascular structure demonstrates high geometric consistency, which is particularly advantageous for standardized modeling and simulation applications that require representative, rather than patient-specific, anatomy.

## Discussion

The presented workflow enables the reconstruction of a geometrically consistent, hollow 3D model of the intracranial arterial system derived from population-based TOF-MRA data. Beyond its role as a purely geometric representation, the model provides a structured framework for anatomical analysis, quantitative evaluation, and downstream clinical and engineering applications.

From an anatomical perspective, the reconstructed geometry allows for the systematic assessment of vessel trajectories, branching patterns, and curvature characteristics of major intracranial arteries. The use of centerline-based reconstruction facilitates a simplified yet anatomically meaningful description of complex 3D vessel courses, enabling comparative analyses across vessel segments. In contrast to purely statistical vascular atlases, the present work explicitly links population-based vascular information to a CAD-ready, manufacturable geometry that can be used directly for simulation and experimental applications. While Dunås et al. focused on probabilistic mapping of major arteries in a cohort of 167 subjects, the current model is derived from a substantially larger cohort of 4308 individuals and is specifically tailored for 3D printing and device-oriented in vitro testing [25, 26]. 

Quantitatively, the model enables the extraction and evaluation of vessel-related metrics such as diameter, length, and curvature along the vascular tree. These parameters are derived from centerline and distance-mapping information and can be analyzed in a spatially resolved manner. As the underlying geometry is based on a large population cohort, the resulting measurements reflect typical anatomical dimensions rather than individual extremes, making the model particularly suitable for standardized analyses and reference-based investigations.

For CAD reconstruction, vessel cross-sections were approximated by circular profiles perpendicular to the local centerline. While this assumption substantially simplifies model generation and ensures robust loft operations, it neglects locally non-circular lumen geometries and shape variations that may occur particularly near bifurcations or curved vessel segments. Consequently, the generated model should be regarded as a geometrically standardized representation rather than an exact anatomical replica.

An additional aspect that merits consideration is the long-term sustainability and interoperability of computational models across disciplines. Standardized model specifications not only facilitate reproducibility within a given research domain, but also lower the barrier for reuse and integration of models in adjacent fields, such as biomedical engineering or clinical decision support. By enabling models to be interpreted, executed, and extended across different software environments and research communities, such standards can foster cumulative model development and accelerate the translation of computational insights into practical and clinical applications [27]. 

A major strength of the presented approach lies in its applicability to clinical and preclinical use cases. The generated hollow vessel model provides a physically and digitally consistent representation of the intracranial vasculature and is immediately suitable for in vitro investigations. In particular, it allows for the simulation and experimental reproduction of neurovascular interventions, including the deployment of catheters, stents, and coils. Under controlled and reproducible anatomical conditions, device-related handling parameters such as pushability and trackability can be systematically investigated, independent of patient-specific variability [28, 29]. 

In addition to physical experimentation, the digital vessel model can serve as a geometric basis for computational applications, including blood flow simulations, stenosis analysis, or virtual device testing. The separation of image processing, CAD-based modeling, and application-specific analysis ensures a modular workflow that remains flexible for future extensions. This includes the integration of advanced material models, hemodynamic simulations, or the adaptation of the pipeline toward patient-specific reconstructions.

Deep-learning methods have achieved promising results in the segmentation of major cerebral arteries and intracranial aneurysms from MRA. These approaches include U-Net-based architectures, residual and dual-attention networks, dilated residual networks, and context-aware preprocessing strategies for reducing the volume of interest. Their principal objective is the automated delineation or volumetric quantification of vascular structures in individual examinations. In contrast, the present study does not propose a new segmentation network. Its contribution lies in the downstream transformation of population-averaged vascular information into a continuous, CAD-compatible, hollow, and physically manufacturable reference geometry. In future implementations, validated deep-learning models could be integrated as an alternative front end for vessel segmentation, provided that they are adapted to the imaging characteristics of population-averaged TOF-MRA data [30–35]. 

The principal contribution of the present study is the transformation of a large population-derived angiographic dataset into a physically manufacturable intracranial reference model. Unlike patient-specific models, which reflect individual anatomy and therefore exhibit considerable geometric variability, the proposed model aims to represent common anatomical characteristics observed across a large population. This approach is particularly advantageous for applications requiring standardized testing environments, such as early-stage device development, methodological benchmarking, educational training, and comparative in vitro investigations. Conversely, the model is not intended for patient-specific treatment planning, prediction of individual intervention outcomes, or assessment of rare anatomical variants.

The present study primarily focuses on the generation of a standardized, population-based intracranial vascular geometry and the establishment of a reproducible reconstruction workflow. Although the resulting model may serve as a basis for future neurointerventional simulations, device testing, educational applications, and computational flow analyses, these applications were not directly validated within the scope of the current work. Their implementation requires additional considerations beyond geometry alone, including material selection, wall compliance, surface properties, manufacturing accuracy, and physiologically realistic flow conditions.

## Limitations

The presented approach has several limitations. Firstly, the use of an averaged TOF-MRA dataset inevitably leads to a loss of very small and low-contrast vessels, particularly distal branches with diameters approaching the voxel size. As a result, the model primarily represents the central and proximal intracranial arteries, while distal branches and perforators are underrepresented or absent. Furthermore, due to the relatively low isotropic resolution of 1 mm and the averaging across a large number of datasets, anatomical variations are smoothed out, leading to the emergence of only a single representative anterior cerebral artery. Consequently, the model is primarily suitable for applications focusing on the middle cerebral artery and vertebral artery.

Secondly, only one side of the intracranial vascular system was modelled in detail. This simplification was chosen to reduce geometric complexity and facilitate handling in experimental setups, but it limits the representation of bilateral anatomical variations and may not reflect asymmetries observed in clinical practice [36]. 

Thirdly, the segmentation and centerline extraction workflow still requires manual interaction, particularly for correcting discontinuities, separating closely adjacent vessels, and removing artifacts near the skull base. Residual segmentation errors and smoothing effects may locally alter vessel diameter and curvature, despite the large underlying cohort and averaging procedure. Furthermore, the hollow vessel model was designed with a uniform wall thickness of 2 mm for manufacturing robustness rather than anatomical accuracy, and thus does not represent physiological wall structure or material properties. Finally, no formal validation against patient-specific geometries or ex vivo vascular casts was performed within this study; future work should include systematic comparisons between the reference model and individual patient anatomies, as well as targeted in vitro experiments, to further quantify geometric fidelity and application-specific suitability.

No formal validation was performed regarding catheter navigation, stent deployment, hemodynamic simulations, or training applications. Consequently, the present work demonstrates the feasibility of generating a standardized vascular reference model rather than validating specific neurointerventional use cases. Future studies should systematically investigate anatomical accuracy, manufacturing fidelity, device interaction, and flow-related behavior under controlled experimental conditions.

The generalizability of the proposed workflow to external datasets remains to be evaluated, as differences in imaging protocols, scanner configurations, and image quality may affect the resulting vascular geometry. Furthermore, the present study focused on model generation rather than application-specific validation. Therefore, additional studies are required to assess the suitability of the model for clinical, educational, and experimental use cases.

The conceptual workflow is expected to be transferable to other angiographic datasets; however, the current implementation was developed using a population-averaged TOF-MRA dataset obtained with a standardized 1.5-T acquisition protocol. Variations in field strength, spatial resolution, voxel anisotropy, slab coverage, scanner manufacturer, reconstruction method, flow-related signal loss, and vessel-to-background contrast may influence vessel enhancement, threshold selection, segmentation continuity, and diameter estimation. The empirically selected segmentation thresholds should therefore be regarded as dataset-specific rather than universally applicable. Application to external datasets will require protocol-specific intensity normalization, parameter adjustment, and independent validation against expert reference segmentations. In particular, validation on native 1.5-T and 3-T TOF-MRA data is needed before generalizability can be claimed.

Potential clinical integration should be considered at different levels. In its current form, the model may support standardized benchtop testing, procedural training, comparison of devices, and technical optimization of neurointerventional workflows. It may also provide a reproducible geometry for computational fluid dynamics and virtual device deployment. However, clinical translation would require application-specific validation, including assessment of manufacturing tolerances, wall compliance, surface friction, pulsatile flow behavior, and compatibility with fluoroscopy, CT, MRI, or navigation systems. Because the model represents averaged anatomy and does not retain patient-specific vascular variants or pathological lesions, it is not intended for individual treatment planning or prediction of patient outcomes.

## Conclusion

With respect to clinical relevance, the presented model should be understood as a reference geometry rather than a substitute for patient-specific anatomy. Its population-based nature enables reproducible and generalizable investigations, which are particularly valuable for comparative studies, methodological validation, and early-stage device development. By providing a CAD-compatible and 3D-printable representation of typical intracranial arterial anatomy, the workflow demonstrates that large-scale angiographic datasets can be transformed into technically usable and anatomically meaningful vascular models, supporting the translation of imaging data into simulation, training, and experimental environments.

## Data Availability

The data that support the findings of this study are based on the SHIP cohort. Access to these data is subject to data protection regulations and is not publicly available. Data may be obtained from the SHIP data management unit upon reasonable request and with permission of the relevant authorities.
